# A murine mesenchymal stem cell model for initiating events in osteosarcomagenesis points to CDK4/CDK6 inhibition as a therapeutic target

**DOI:** 10.1038/s41374-021-00709-z

**Published:** 2021-12-17

**Authors:** Natasja Franceschini, Raffaele Gaeta, Paul Krimpenfort, Inge Briaire-de Bruijn, Alwine B. Kruisselbrink, Karoly Szuhai, Ieva Palubeckaitė, Anne-Marie Cleton-Jansen, Judith V. M. G. Bovée

**Affiliations:** 1 Department of Pathology, Leiden University Medical Center, Leiden, The Netherlands; 2 Department of Translational Research and New Technologies in Medicine and Surgery, University of Pisa, Pisa, Italy; 3 Division of Molecular Genetics, The Netherlands Cancer Institute, Amsterdam, The Netherlands; 4 Department of Cell and Chemical Biology, Leiden University Medical Center, Leiden, The Netherlands

**Keywords:** Sarcoma, Cancer genetics, Cancer models, Targeted therapies

## Abstract

Osteosarcoma is a high-grade bone-forming neoplasm, with a complex genome. Tumours frequently show chromothripsis, many deletions, translocations and copy number alterations. Alterations in the p53 or Rb pathway are the most common genetic alterations identified in osteosarcoma. Using spontaneously transformed murine mesenchymal stem cells (MSCs) which formed sarcoma after subcutaneous injection into mice, it was previously demonstrated that p53 is most often involved in the transformation towards sarcomas with complex genomics, including osteosarcoma. In the current study, not only loss of p53 but also loss of p16^Ink4a^ is shown to be a driver of osteosarcomagenesis: murine MSCs with deficient p15^Ink4b^, p16^Ink4a^, or p19^Arf^ transform earlier compared to wild-type murine MSCs. Furthermore, in a panel of nine spontaneously transformed murine MSCs, alterations in p15^Ink4b^, p16^Ink4a^, or p19^Arf^ were observed in eight out of nine cases. Alterations in the Rb/p16 pathway could indicate that osteosarcoma cells are vulnerable to CDK4/CDK6 inhibitor treatment. Indeed, using two-dimensional (*n* = 7) and three-dimensional (*n* = 3) cultures of human osteosarcoma cell lines, it was shown that osteosarcoma cells with defective p16^INK4A^ are sensitive to the CDK4/CDK6 inhibitor palbociclib after 72-hour treatment. A tissue microarray analysis of 109 primary tumour biopsies revealed a subset of patients (20–23%) with intact Rb, but defective p16 or overexpression of CDK4 and/or CDK6. These patients might benefit from CDK4/CDK6 inhibition, therefore our results are promising and might be translated to the clinic.

## Abstract (web summary)

Osteosarcoma is a tumour with a highly complex genome, which hampers the identification of driver genes. Using a model of murine mesenchymal stem cells (MSCs) with deficient p15^Ink4b^, p16^Ink4a^, or p19^Arf^ that transform earlier compared to wild-type MSCs, the authors demonstrated that loss of p16^Ink4a^ is a driver of osteosarcomagenesis. This can be exploited with a CDK4/CDK6 inhibitor, as osteosarcoma cells showed sensitivity to palbociclib which might be used as a novel therapeutic option.

## Introduction

Osteosarcoma is the most common malignant mesenchymal tumour of the bone in children and adolescents and characterized by osteoid formation. Compared to other cancer types driven by a specific mutation, osteosarcomas show highly complex genomes with a relatively high occurrence of chromoanagenesis, such as chromothripsis^1–3^. Recurrent alterations are rare, but most often osteosarcomas harbour loss-of-function alterations in *TP53* (47–90%)^1,4,5^. The second most common alteration inactivates *RB1* (29–47%)^4,5^, a tumour suppressor gene controlling cell cycle progression^6^. Other regulators of the cell cycle pathway are also often affected in osteosarcoma. *CDK4* is amplified in 10% of high-grade osteosarcomas, and together with *CDK6* directly controls Rb activity by phosphorylation of Rb^7–9^. Upstream of the Rb pathway, p15^INK4B^ and p16^INK4A^ can inhibit CDK4 and CDK6 activity^6^. p15^INK4b^ is transcribed from the *CDKN2B* gene, whereas p16^INK4A^, and its alternate reading frame p14^ARF^ (p19^Arf^ in mouse), is transcribed from the *CDKN2A* gene (a schematic overview of the locus is depicted in Supplementary Fig. S1). p14^*ARF*^/p19^Arf^ is involved in activating p53-dependent growth arrest^10^. *CDKN2B* and *CDKN2A* are adjacent loci on the genome and are often co-deleted. Somatic alterations in both genes have been identified in 14–19% of osteosarcomas and also often in other tumour types^11,12^. In particular, deletions of *CDKN2A* are clinically relevant, as loss of p16^INK4A^ is correlated with poor overall survival and poor response to chemotherapy^13–18^.

We previously demonstrated that murine and canine mesenchymal stem cells (MSCs) spontaneously transform in vitro and can be used to model driver or initiating events involved in the development of sarcomas with complex genomics, including osteosarcoma^19^. We showed that spontaneously transformed murine MSCs harbour point mutations in *Trp53* and/or copy number alterations in *Cdkn2a* and *Cdkn2b*. Upon inactivation of *Trp53*, murine MSCs transformed earlier compared to wild-type, confirming the contribution of loss of p53 to spontaneous transformation and development of sarcomas with a complex genome.

In the current study, we investigate the role of the *Cdkn2a/Cdkn2b* genes in the spontaneous transformation of murine MSCs towards osteosarcoma. We show that murine MSCs with deficient p15^Ink4b^, p16^Ink4a^, or p19^Arf^ transform earlier compared to wild-type MSCs. Furthermore, we demonstrate that the defective cell cycle regulation pathway caused by p16^INK4A^ inactivation can be therapeutically exploited using the selective CDK4/CDK6 inhibitor palbociclib in both 2D and 3D in vitro culture models of osteosarcoma cell lines. Our study demonstrates that 20–23% of primary osteosarcoma biopsies showed intact Rb, but defective p16 ^INK4A^ or overexpression of CDK4 and/or CDK6, indicating potential benefit from CDK4/CDK6 inhibition in almost one quarter of osteosarcoma patients.

## Materials and methods

### Cell culture

Murine bone-marrow derived mesenchymal stem cells (MSCs) were isolated as described previously^19^, from surplus C57BL/6 J (B6_4, B6_5, B6_7, B6_10), surplus NMRI (NMRI_2, NMRI_3, NMRI_9) mice, or C57BL/6 J mice kindly gifted by Dr. Melissa van Pel (BM42, BM91). Growth curves, differentiation capacity, in vivo growth capacity and a detailed genomic analysis using whole genome sequencing have been described elsewhere for B6_4, B6_7, B6_10^19^. Additional murine MSCs were isolated from surplus FVB mice and mice with deficient p15^Ink4b^ (Ink4b^−/−^), p16^Ink4a^ (Ink4a^−/−^), p15^Ink4b^ and p16^Ink4a^ (Ink4ab^−/−^), or p19^Arf^ (Arf^−/−^) mice. Mice with deficient p15^Ink4b-^ (Ink4b^−/−^) and p16^Ink4a^ (Ink4a^−/−^) were generated as described previously^20^ (Supplementary Fig. S1). Genetic knockout was confirmed at the protein level by Western blotting and for Ink4ab^−/−^ mice also at the DNA level by PCR using 5′GCAGTGTTGCAGTTTGAACCC 3′ as a forward primer and 5′TGTGGCAACTGATTCAGTTGG 3′ as a reverse primer^20^. All murine MSCs were cultured in alpha MEM (Gibco, Invitrogen Life Technologies, Scotland, UK) supplemented with 15% Performance plus fetal bovine serum (FBS) (Gibco), 1% Pen/Strep (Gibco), and 1% Glutamax (Gibco) at 37 °C with 5% CO_2_ in a humidified incubator and were tested regularly for mycoplasma. Each passage cells were trypsinized and counted with a Bürker-Türk counting chamber to calculate population doublings.

Human osteosarcoma cell lines 143B, MG63, MHM, SAOS2, ZK58, HAL and KPD were cultured in RPMI 1640 (Gibco), supplemented with 10% FBS, in a humidified incubator at 37 °C and 5% CO_2_. Human cell lines were retrieved from the EuroBoNet consortium^21^ and were regularly STR profiled using the GenePrint 10 system kit (Promega, Madison, WI, USA) and tested for mycoplasma. For the generation of multi-cellular tumour spheroids (MCTS) of osteosarcoma cell lines (protocol adapted from ref. ^22^), cells were suspended in medium combined with methylcellulose (0.24% (w/v) dissolved in DMEM), and seeded into a 1% agarose coated 96-well plate for seven days before the start of an experiment.

### Drug treatment

For 2D cultures, human osteosarcoma cell lines or murine MSCs were seeded (between 3000 and 6000 cells per well) into 96-well plates and after 24 h treated with PBS or Palbociclib (dissolved in PBS, PD-0332991, Selleckchemicals, Houston, TX, USA) in concentrations ranging from 0.01 µM to 100 µM. Cells were fixed three days after treatment with 4% formaldehyde and stained with 2 µg/ml Hoechst (Invitrogen Life Technologies, Thermo Fisher Scientific, MA, USA) and nuclei were counted with the Cellomics ArrayScan VTI HCS 700 and HCS Studio Cell Analysis Software (Thermo Fisher Scientific). For MCTS, cells were treated with PBS or palbociclib in concentrations ranging from 0.1 µM to 100 µM. Cells were incubated three days after treatment with PrestoBlue cell viability reagent (Invitrogen Life Technologies) for 90 min and fluorescence was measured using a microplate reader (Infinite M Plex, Tecan Group Ltd., Zürich, Switzerland). After read-out, MCTS were fixed in 4% formaldehyde containing Alcian Blue (1:400) and paraffin embedded. To determine cell viability relative to PBS control, dose response curves were made using Graphpad Prism 8 software, after correcting for background reads and normalized growth rates of each cell line, as described previously^23^.

### Transformation analysis of mesenchymal stem cells

Late passage (> passage 8) murine MSCs were subjected to metaphase analysis and the soft agar anchorage independent growth assay as described previously^19^. In short, for metaphase harvest murine MSCs were seeded and treated with Calyculin A (80 nM, LC Laboratories, Woburn, MA, USA). Hereafter, cells were washed, incubated with KCl (0.075 M) and fixed with methanol/glacial acetic acid in a ratio of 4:1. The cell suspension was dropped onto a slide and stained with DAPI for microscopic counting of metaphase chromosomes. For the soft agar anchorage independent growth assay, a cell suspension of 50,000 cells were seeded into a top layer of 0.35% agarose, on top of a bottom layer of 0.7% agarose in non-tissue culture treated 6-well plates and incubated in a humidified incubator at 37 °C with 5% CO_2_ for 4 weeks, after which cells were imaged with GelCount (Oxford Optronix, Milton, UK). Previously transformed cells (B6_10)^19^ were taken along as a positive control.

### Western blotting

For p15, p16, Rb and GAPDH Western blots, whole cell Hot-SDS lysates of murine MSCs or osteosarcoma cell lines were collected as described previously^19^. For p19 and Histon H3 western blots, nuclear lysates were made by washing cells twice with cold PBS, followed by the addition of PBS-Triton X (0.5%) for 10 min, while shaking on ice. Cells were centrifuged twice, washed with PBS-Triton X (0.5%) and the pellet was resuspended in Hot-SDS buffer (1% SDS, 10 mM EDTA, 10 mM Tris pH 7.4) containing protease inhibitor cocktail (Roche, Basel, Switzerland) and phosphatase inhibitor cocktail (Roche). Protein concentrations of lysates were determined with the Biorad DC^TM^ protein assay kit (Bio-rad, Hercules, CA, USA) according to the manufacturer’s protocol, measured with a microplate reader (Infinite M Plex, Tecan Group Ltd.).

Sample loading, blotting and quantification were performed as previously described^19^. Blots were stained for p15 (1:500, Abcam, Cambridge, UK), p16 (1:1000, clone JC8, Immunologic, WellMed BV, Duiven, The Netherlands), p19 (1:5000, clone ab80, Abcam), Rb (1:500, clone G3-245, BD Pharmingen, San Diego, CA, USA), Histon H3 (1:1000, Cell Signalling, Leiden, The Netherlands), or GAPDH (1:3000, Cell Signalling). Blots were developed with SuperSignal West Pico PLUS Chemiluminescent Substrate (Thermo Fisher Scientific) using the ChemiDoc Touch Imaging System (Bio-rad).

### Immunohistochemistry

#### Tissue micro array construction

For this study five osteosarcoma tissue micro arrays (TMA) were used, that contain FFPE samples of primary tumour biopsies, primary tumour resections, local relapses and metastases of 158 patients (not all sample types were available for each patient). Clinicopathological details can be found in Supplementary Table S1. Good histological response to chemotherapy was defined as ≥90% tumour necrosis after chemotherapy^24^. The construction of one of the TMAs has been described previously (cohort 2)^25,26^. For the construction of TMAs for cohorts 1,3, and 4, punches (1.0 mm (cohort 3) or 1.5 mm (cohort 1and 4)) of FFPE samples were placed into an acceptor block using the TMA master (3DHISTECH, Budapest, Hungary). For each tumour, three tissue-cores were present in the same block. For cutting sections, the tape transfer system (39475205, Leica Biosystems, Wetzlar, Germany) was used. Each TMA also contained other tissue types as internal controls for immunohistochemistry.

#### Immunohistochemical staining

Slides of each TMA or slides containing sections (4 µm) of paraffin embedded MCTS were stained with haematoxylin and eosin (H&E) or used for immunohistochemical staining after deparaffinisation and rehydration. TMA sections were stained for CDK6 (151213, Abbiotec, San Diego, CA, USA), CDK4 (12790, clone D9G3E, Cell Signaling) or Rb (554136, clone G3245, BD Pharmingen). MCTS sections were stained for Rb, Ki67 (clone D2H10, Cell Signaling), cleaved caspase 3 (Cell Signaling), and SATB2 (clone CL0276, Sigma). For CDK6, SATB2, Ki67 and cleaved caspase 3 staining, antigen retrieval was performed by incubation in 10 mM citrate buffer (pH 6) for 10 min and cooling down for 2 h. For CDK4 and Rb staining, antigen retrieval was performed in Tris-EDTA (pH 9). Sections were incubated with primary antibody (Ki67, 1:1600; cleaved caspase 3, 1:800; CDK4, 1:4000; CDK6, 1:100; Rb, 1:2000, SATB2, 1:10) overnight at 4 °C. The next day, sections were incubated with BrightVision one step detection system poly-HRP anti-mouse/rabbit (VWRKDPVO110HRP, Immunologic) for 30 min. Sections were then washed with PBS and DAB+ Chromogen (K3468, Dako, Agilent Technologies, CA, USA) was added to each slide for 10 min. Slides were counterstained with haematoxylin, dehydrated and mounted.

#### Immunohistochemical scoring

For TMAs two independent observers (N.F. and R.G.) scored each core semi-quantitatively based on the intensity of the staining and percentage of positive cells, as described previously^27^. For intensity, a value of 1 (weak), 2 (moderate), or 3 (strong) was given, and for the percentage of positive cells, a value of 1 (0–24%), 2 (25–49%), 3 (50–74%), or 4 (75–100%). For CDK4 and Rb the staining was nuclear, but for CDK6 both nuclear and cytoplasmic staining was evaluated. Tumours were regarded as positive if the sum of scores for intensity and percentage of positive cells was ≥4 for CDK4^27^. Tumours were classified as CDK6 high when the sum of cytoplasmic and nuclear scores for CDK6 was ≥7^28^. Tumours with a sum of scores equal to 0, yet with a positive internal control, were regarded as negative for Rb staining. In case of discrepancies between the two observers, slides were reviewed by a third observer (J.V.M.G.B.) to reach a consensus. For quantification of MCTS, the number of positive cells of cleaved caspase 3 and Ki67 stainings was determined using QuPath Software v.0.2.3 on three different sections on each slide^29^.

### Statistical analysis

For statistical comparisons between two groups, a Student’s *t* test was performed. Correlation analysis was performed using Spearman’s correlation. Comparisons between survival curves were performed using the Log-rank (Mantel–Cox) test. All statistical analyses were performed using GraphPad Prism v.8. Comparisons were considered statistically significant using a significance level of 5%.

## Results

### Loss of *CDKN2A/CDKN2B* is frequent in spontaneously transformed murine MSCs

**Fig. 1:**
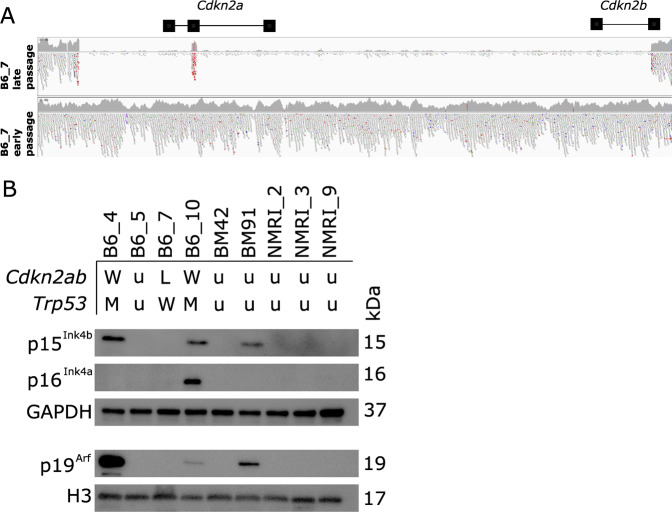
Transformed murine MSCs of different mouse strains frequently show loss of protein expression of p15^Ink4b^, p16^Ink4a^ and p19^Arf^. **A** IGV viewer showing a homozygous deletion including both *Cdkn2a* and *Cdkn2b* genes in transformed (late passage) murine MSC B6_7. Early passage cells (P2) were collected prior to the transformation event, whereas late passage cells (P15) were collected after spontaneous transformation. **B** Western blot depicting protein expression of p15^Ink4b^, p16^Ink4a^, p19^Arf^ in transformed murine MSCs from C57BL/6 J (B6_4, B6_7, B6_10, BM42, BM91) or NMRI mice (NMRI_2, NMRI_3, NMRI_9). GAPDH or Histon H3 was used as a loading control. The size of each band is indicated in kDa. The genomic status of genes *Cdkn2a*, *Cdkn2b*, and *Trp53* is indicated for transformed murine MSCs as described previously^19^. M = point mutation, W = wildtype, L = homozygous loss, u = no genomic status available.

We previously identified a large deletion in the *Cdkn2a/Cdkn2b* locus in one of three transformed murine MSC cultures (B6_7), that formed sarcoma in vivo^19^, for which the *Cdkn2a/Cdkn2b* locus is shown in Fig. 1A. To evaluate the relevance of *Cdkn2a* and *Cdkn2b* loss we used a larger panel of nine spontaneously transformed murine MSCs, originating from different strains (C57BL/6 J or NMRI), for expression of p15^Ink4b^, p16^Ink4a^ and p19^Arf^ at the protein level. Transformation of six out of nine murine MSC lines have been described elsewhere (B6_4, B6_7, B6_10, NMRI_2, NMRI_3, NMRI_9)^19^. For the additional three lines, the transformation event was confirmed by karyotyping (B6_5, BM42, BM91; Supplementary Fig. S2). Transformed murine MSC line B6_7 indeed showed loss of protein expression of p15^Ink4b^, p16^Ink4a^ and p19^Arf^. Furthermore, loss of p15^Ink4b^, p16^Ink4a^ or p19^Arf^ is a common event, as six out of nine MSCs have lost p15^Ink4b^ expression, and six out of nine MSCs have lost p19^Arf^ expression (Fig. 1B). Eight out of nine MSCs show loss of p16^Ink4a^ expression. However, in this Western blot loss of p16^Ink4a^ protein expression is not always indicative for p16^Ink4a^ deletion on genomic level, illustrated for instance by murine MSC line B6_4 that has intact p16^Ink4a^ based on whole-genome sequencing data (Fig. 1B)^19^, but does not show p16^Ink4a^ protein expression.

### Murine MSCs deficient for p16^Ink4a^ and deficient for p15^Ink4b^ transform earlier compared to wildtype MSCs

**Fig. 2:**
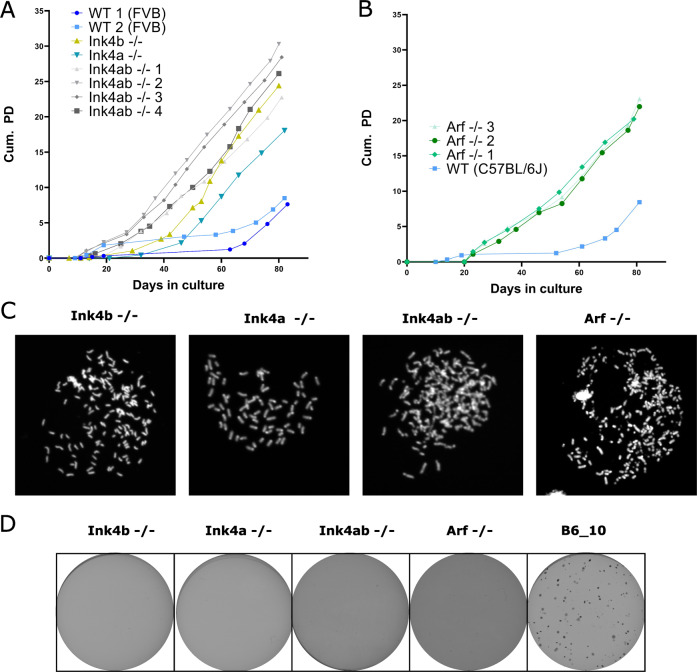
Earlier transformation of p16 or p15 knockout MSCs. Murine MSCs isolated from **A** Ink4a^−/−^ (*n* = 1), Ink4b^−/−^ (*n* = 1), Ink4ab^−/−^ (*n* = 4) **B** or Arf^−/−^ (*n* = 3) transform earlier compared to wild-type MSCs from FVB or C57BL/6 J mice, based on cumulative population doublings (Cum. PD.). Each datapoint represents a passage of the cell culture. **C** Metaphase analysis of murine MSCs from Ink4a^−/−^, Ink4b^−/−^, Ink4ab^−/−^, or Arf^−/−^ mice showed abnormal chromosome numbers, higher than the normal modal number of 40. **D** Soft agar anchorage independent growth assay of murine MSCs from Ink4a^−/−^, Ink4b^−/−^, Ink4ab^−/−^, or Arf^−/−^ mice showed no colony formation. Transformed murine MSCs from B6_10 were used as a positive control.

To evaluate whether loss of p16^Ink4a^ and p15^Ink4b^ or both is important in the spontaneous transformation of murine MSCs, MSCs from mice with loss of p16^Ink4a^ (Ink4a^−/−^), loss of p15^Ink4b^ (Ink4b^−/−^), or both (Ink4ab^−/−^) were cultured long-term to observe when transformation would occur (for a schematic overview of the knockout mice used in this study see Supplementary Fig. S1). Murine MSCs from Ink4a^−/−^, Ink4b^−/−^ or Ink4ab^−/−^ mice transformed earlier (after 25-46 days) compared to wild-type murine MSCs (after 64-76 days) (Fig. 2A). As the *Cdkn2a* gene also encodes an alternative reading frame, p19^Arf^, murine MSCs with a loss of p19^Arf^ were cultured and also shown to transform earlier (after 23 days) compared to wildtype MSCs (after 61 days) (Fig. 2B). For all knockout mice, the knockout was confirmed by Western blotting and for mice with deficient p16^Ink4a^; p15^Ink4b^ (Ink4ab^−/−^) mice also at the DNA level by PCR (Supplementary Fig. S3). Transformation of murine MSCs was confirmed by metaphase analysis (Fig. 2C), as late passage MSCs had higher than the normal modal number of 40 chromosomes. However, none of the knockout MSCs showed soft agar anchorage independent growth (Fig. 2D). These results show that loss of p16^Ink4a^ and/or p15^Ink4b^ enhanced proliferation and genomic alterations suggesting transformation.

### Transformed murine MSCs with loss of p16^Ink4a^ and p15^Ink4b^ are sensitive to palbociclib

**Fig. 3:**
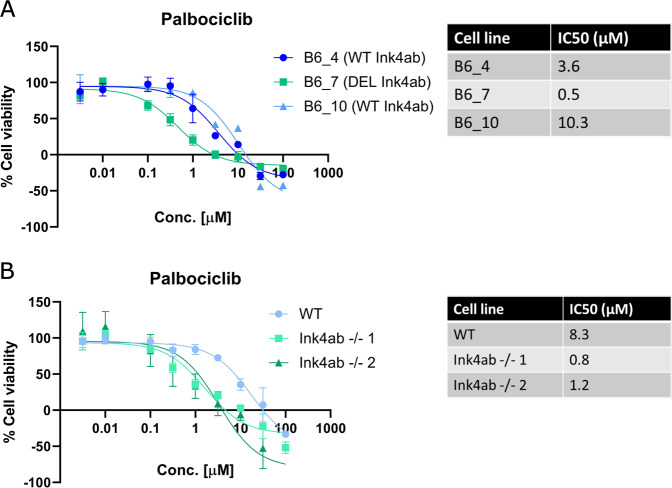
Transformed murine MSCs are sensitive to CDK4/CDK6 inhibitor palbociclib. Murine MSCs (**A**) that have spontaneously transformed after long-term culture (B6_4, wildtype (WT) Ink4ab; B6_7, deletion (DEL) of Ink4ab; B6_10, wildtype (WT) Ink4ab) or **B** MSCs from Ink4ab^−/−^ mice after transformation were treated with palbociclib for 72 h after which relative cell viability and IC_50_ values were determined. Wildtype MSCs were derived from FVB mice. Data points represent the mean of three experiments performed ± standard deviation.

Within the Rb pathway, the Ink4 proteins inhibit CDK4/CDK6 activity, thereby inhibiting cell cycle progression. Therefore loss of p16^Ink4a^ and p15^Ink4b^ could make cells more vulnerable to CDK4/CDK6 inhibitors to decrease uncontrolled cell proliferation. To investigate this in our murine mesenchymal stem cell model, transformed murine MSCs, in which p16^Ink4a^ and p15^Ink4b^ loss was confirmed, were treated with the CDK4/CDK6 inhibitor palbociclib. Murine MSCs in which whole-genome sequencing previously confirmed loss of p16^Ink4a^ and p15^Ink4b^ (B6_7)^19^ were more sensitive to palbociclib, with an IC_50_ of 0.5 µM compared to transformed MSCs that have intact p16^Ink4a^ and p15^Ink4b^ (B6_4 and B6_10), with IC_50_ values of 3.6 and 10.3 µM, respectively (Fig. 3A). To determine whether the increased sensitivity was caused by loss of p16^Ink4a^ and p15^Ink4b^, murine MSCs isolated from Ink4ab^−/−^ mice were treated with palbociclib (Fig. 3B). MSCs from Ink4ab^−/−^ mice showed the highest sensitivity to palbociclib, with IC_50_ values between 0.8 and 1.2 µM, compared to wild-type MSCs, with an IC_50_ of 8.3 µM, suggesting that loss of p16^Ink4a^ and p15^Ink4b^ increases sensitivity to palbociclib.

### Human osteosarcoma cell lines are sensitive to palbociclib

**Fig. 4:**
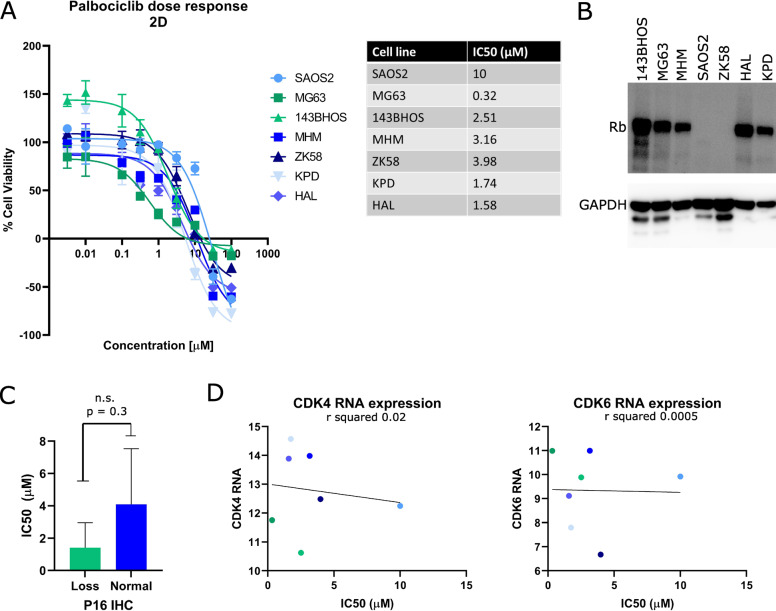
2D cultures of human osteosarcoma cell lines are sensitive to CDK4/CDK6 inhibitor palbociclib. **A** Osteosarcoma cell lines were treated with palbociclib for 72 h after which relative cell viability and IC_50_ values were determined. Cell lines MG63 and 143BHOS (green) have loss of p16, whereas other OS cell lines have intact p16 (blue). Data points represent the mean of three experiments performed ± standard deviation. **B** Western blot showing expression levels of Rb in osteosarcoma cell lines. GAPDH was used as a loading control. **C** p16 protein expression status of each osteosarcoma cell line as published^21^ was correlated with IC_50_ values from the current study. n.s. not statistically significant. **D** CDK4 and CDK6 RNA expression levels as published elsewhere^30^ of each cell line did not correlate with IC_50_ values from the current study. Each dot represents one cell line.

Osteosarcoma cell lines were treated with palbociclib in 2D cultures and IC_50_ values were determined (Fig. 4A). A highly variable dose-dependent response to palbociclib in all osteosarcoma cell lines was observed. As the efficacy of CDK4/CDK6 inhibition relies on the presence of intact Rb, the Rb status of each osteosarcoma cell line was determined by Western blot. All osteosarcoma cell lines (143B, MG63, MHM, HAL and KPD), except for SAOS2 and ZK58, showed Rb expression (Fig. 4B). SAOS2 and ZK58, with loss of Rb, indeed showed the highest IC_50_ of palbociclib compared to other osteosarcoma cell lines. However, the difference in IC_50_ value between ZK58 and other osteosarcoma cell lines with intact Rb was smaller than for SAOS2. The expression status of other proteins involved in the p16-Rb pathway was investigated, including p16^INK4A^, CDK4 and CDK6. p16^INK4A^ protein expression of each cell line was based on immunohistochemical expression published previously^21^ (Fig. 1B). The p16 immunohistochemical status of each cell line was combined with the IC_50_ values from the current study. Although osteosarcoma cells with loss of p16^INK4A^ showed on average a lower IC_50_ to palbociclib (1.4 µM) than cells with normal p16^INK4A^ expression (4.1 µM), this difference was not statistically significant (*p* = 0.3) (Fig. 4C). *CDK4* and *CDK6* RNA expression levels were derived from a previously published dataset^30^, and correlation analysis was performed using IC_50_ values from the current study. There was no correlation between *CDK4* and *CDK6* RNA expression and sensitivity to palbociclib in osteosarcoma cell lines (Fig. 4D).

### 3D cultures of osteosarcoma are also sensitive to palbociclib

**Fig. 5:**
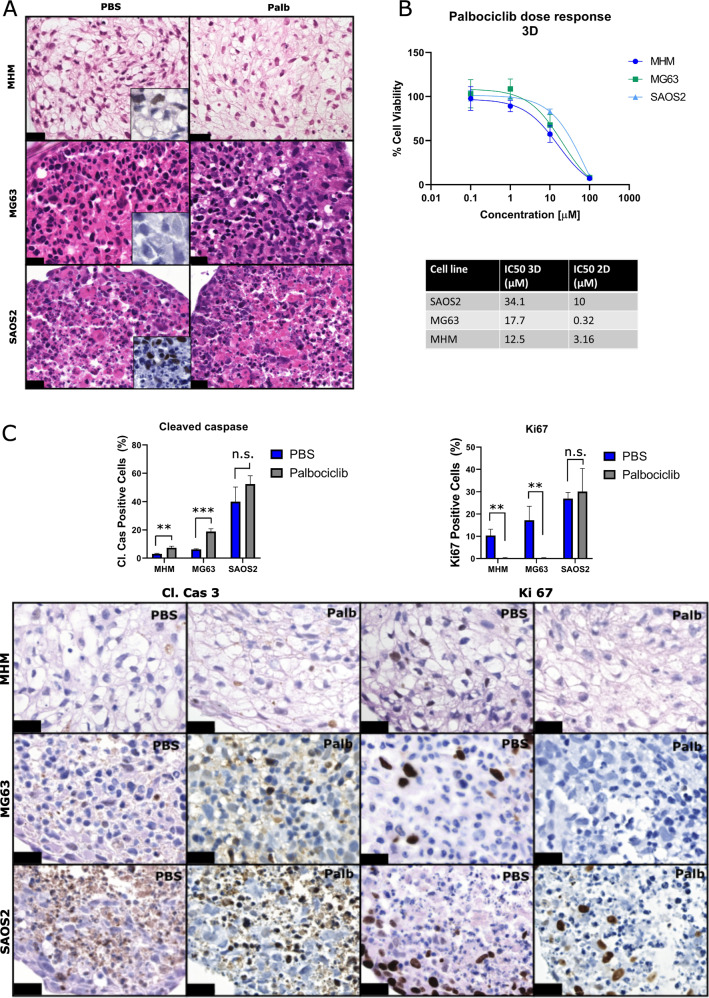
3D cultured MCTS of osteosarcoma cell lines are also sensitive to palbociclib. **A** Hematoxylin and eosin staining of osteosarcoma MCTS treated with palbociclib for 72 h. Scalebar represents 20 µm. Inset shows SATB2 staining. **B** Relative cell viability and IC_50_ values were determined after treatment with palbociclib. **C** Cleaved caspase 3 and Ki67 staining and quantification of 3D cultured MCTS of OS cell lines treated with 10 µM palbociclib (Palb) or PBS. Scale bar represents 20 µm. n.s. not statistically significant, **p ≤ 0.01, ***p ≤ 0.001.

Since 2D cultures might be less representative for the in vivo situation compared to 3D cultures, as demonstrated in previous studies^31–33^, the response to palbociclib in 3D cultures of osteosarcoma cell lines was investigated. Multi-cellular tumour spheroids (MCTS) of three osteosarcoma cell lines were generated (MHM, SAOS2 and MG63) and histological analysis showed the morphological heterogeneity that is also seen in primary tumours (Fig. 5A). MCTS of SAOS2 and MHM show areas suggestive of extracellular matrix deposition and tumour cells were focally positive for SATB2, a marker for osteogenic differentiation (Fig. 5A). MCTS of all osteosarcoma cell lines showed a dose-dependent decrease in cell viability after treatment with palbociclib (Fig. 5B). The IC_50_ values of 3D cultures were higher compared to 2D cultures (Fig. 5B). In MCTS of osteosarcoma cell lines MHM and MG63, palbociclib treatment significantly reduced the percentage of proliferating cells and increased apoptosis as evident from Ki67 and cleaved caspase 3 staining, respectively (Fig. 5C).

### Protein expression of Rb, CDK4 and CDK6 in osteosarcoma patient primary tumour tissue

**Fig. 6:**
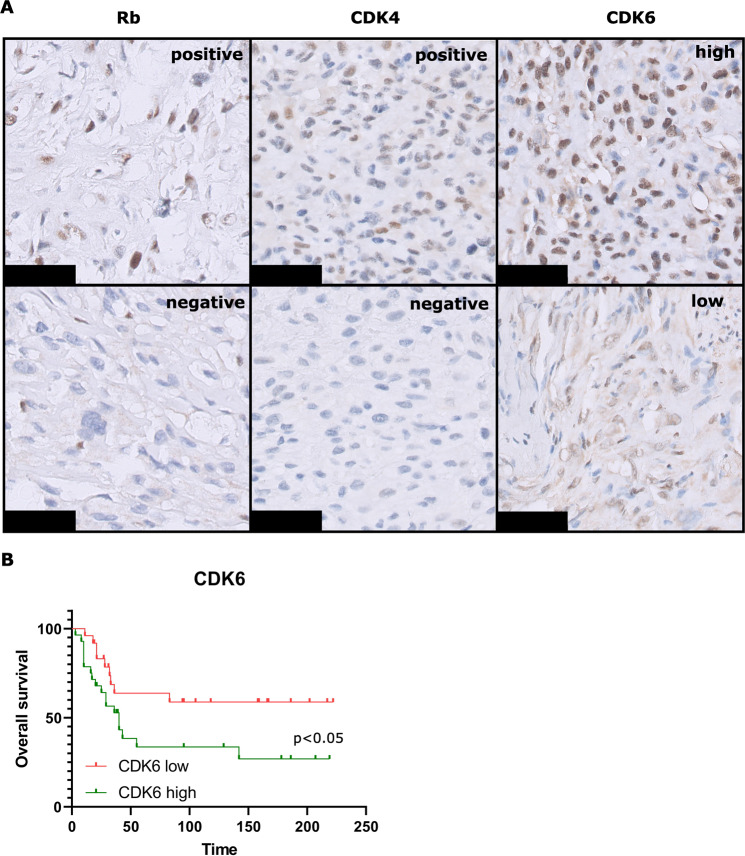
Immunohistochemistry of osteosarcoma tissue micro array. **A** Example of immunohistochemical staining of Rb, CDK4, or CDK6, scored as positive or negative in tissue micro-arrays of osteosarcoma. Scale bar represents 50 µm. **B** Kaplan–Meier curves of overall survival in osteosarcoma patients, based on CDK6 scores.

To estimate which percentage of osteosarcoma patients might be eligible for palbociclib treatment, the expression levels of proteins in the p16-Rb pathway, including Rb, CDK4, CDK6 and p16, was determined in primary tumour tissue of 109 patients using TMA (Table 1; Fig. 6A). As Rb status determines the efficacy of CDK4/CDK6 inhibition^34,35^, first Rb expression was evaluated. 36.4% of the osteosarcomas have lost expression of Rb. As palbociclib directly inhibits CDK4 and CDK6, protein expression levels of CDK4 and CDK6 were determined. 23.7% of primary tumours were CDK4 positive and 44.8% were CDK6 high. p16 scores have been determined in a previous study in osteosarcoma patients (cohort 2), where 20.5% of osteosarcoma patients showed loss of p16^13^. As a combination of Rb expression and CDK4/CDK6 expression or loss of p16 expression are expected to imply sensitivity to CDK4/CDK6 inhibition, the combination scores were determined (Table 1). Of the primary tumour biopsies, 22.7% of the tumours were positive for both Rb and CDK4, 52.9% were positive for Rb and CDK6, and 43.1% was positive for Rb, and CDK4 or CDK6. In cohort 2, 13.3% of the primary tumour biopsies were positive for Rb, but lost p16 expression. In total, between 20.0% and 23.3% of the tumours are Rb^positive^/p16^negative^, Rb^positive^/CDK4^positive^, or Rb^positive^/CDK6^high^, which corresponds to the group of patients that might benefit from CDK4/CDK6 inhibitor treatment. A previous study showed that p16 loss was prognostic for poor overall survival in osteosarcoma patients^13^. In the current study, CDK6 scores were found to be prognostic for overall survival, where patients with overexpression of CDK6 have a worse overall survival compared to those patients with low CDK6 expression (Fig. 6B). For Rb and CDK4 expression there was no significant difference in survival (Supplementary Fig. S4). Neither Rb, CDK4, or CDK6 scores are prognostic for metastasis-free survival (Supplementary Fig. S4) or response to chemotherapy (not shown).

**Table 1:** Overview of immunohistochemical expression of Rb, CDK4 and CDK6, combined with previously published^13^ p16 protein expression results, in tissue micro-arrays of osteosarcoma.

RB	Primary tumour biopsy	Primary tumour resection	Local recurrence	Metastasis
	Total (*n* = 66)	Total (*n* = 68)	Total (*n* = 9)	Total (*n* = 38)
positive	42 (63.6%)	42 (61.7%)	6 (66.7%)	32 (84.2%)
negative	24 (36.4%)	26 (38.2%)	3 (33.3%)	6 (15.8%)
**CDK4**	**Total (*n*** = **76)**	**Total (*n*** = **77)**	**Total (*n*** = **9)**	**Total (*n*** = **38)**
positive	18 (23.7%)	15 (19.5%)	4 (44.4%)	7 (18.4%)
negative	58 (76.3%)	62 (80.5%)	5 (55.6%)	31 (81.6%)
**CDK6**	**Total (*n*** = **58)**	**Total (*n*** = **56)**	**Total (*n*** = **7)**	**Total (*n*** = **26)**
high	26 (44.8%)	32 (57.1%)	6 (85.7%)	12 (46.2%)
low	32 (56.2%)	24 (42.8%)	1 (14.3%)	14 (53.8%)
**Combination status**				
RB^positive^ and CDK4^positive^	15/66 (22.7 %)			
RB^positive^ and CDK6^positive^	27/51 (52.9%)			
RB^positive^ and CDK4^positive^ and CDK6^positive^	6/51 (11.8%)			
RB^positive^ and CDK4^positive^ or CDK6^positive^	22/51 (43.1%)			
**P16 (combination) status**				
RB^positive^ and p16^negative^	4/30 (13.3%)			
RB^positive^ /p16^negative^ or RB^positive^ /CDK4^positive^	6/30 (20.0%)			
RB^positive^ /p16^negative^ or RB^positive^ /CDK6^positive^	7/30 (23.3%)

## Discussion

Transformed murine MSCs provide an excellent model to identify drivers of transformation in osteosarcoma. In addition to p53 alterations^19^, transformed murine MSCs also frequently lose expression of p15^Ink4b^, p16^Ink4a^ and p19^Arf^. However, the protein expression status did not always correspond to the genomic status, as is evident for MSC line B6_4 with loss of p16^Ink4a^ protein expression without concomitant genomic alterations at the *CDKN2A* locus. Alternative mechanisms of p16^Ink4a^ protein expression loss, such as promotor methylation may be active. Nevertheless, the loss of expression of p15^Ink4b^, p16^Ink4a^ and p19^Arf^ in murine MSCs, reflects the genomic status of human osteosarcoma where alterations in p15^INK4B^, p16^INK4A^, or p14^ARF^ are also frequently found^4,5,7,8,25^.

This study shows that loss of either p15^Ink4b^, p16^Ink4a^, or p19^Arf^ in the murine MSC model accelerates transformation. Using the same murine MSC model, we have previously published that these transformed murine MSCs, including those with loss of *CDKN2A/CDKN2B*, show hallmarks of osteosarcoma with a highly complex genome and many copy number alterations^19^. Although chromothripsis could not be evaluated in the current study, not all human osteosarcomas show signs of chromothripsis either, since this only occurs in ~30% of cases^2^. In another study, we have demonstrated that mice injected with transformed murine MSCs, all with loss of *CDKN2A/CDKN2B*, formed osteosarcoma, with evident osteoid formation by atypical tumour cells^13^. Thus, our results combined with previously published results, suggest that loss of *CDKN2A/CDKN2B* is an early driver event in osteosarcoma. Loss of genes in the Rb-p16 pathway is indeed considered as an early event in the transformation towards osteosarcoma^7,36,37^. The exact interplay with alterations in the p53 pathway is currently unknown, and since not all late passage murine MSCs with known loss of p16^Ink4a^ or p53 could form colonies in soft agar, it is not yet known which combination of genomic alterations are required for transformation. This would warrant further investigation.

Loss of p16^INK4A^ protein expression as a result of homozygous loss of the *CDKN2A* genomic region has previously been shown to correlate with poor prognosis as well as a poor response to neoadjuvant chemotherapy in osteosarcoma patients^13–18,25,38^. Thus, studying the loss of p16^INK4A^ in osteosarcoma is clinically relevant and gives a rationale for exploring the Rb-p16 pathway as a novel therapeutic option. Cells with loss of p16^INK4A^ are hypothesized to be more sensitive to CDK4/CDK6 inhibition. Therefore, we investigated the sensitivity to CDK4/CDK6 inhibitor palbociclib in osteosarcoma cells. We confirmed that both 2D and 3D cultures of osteosarcoma cells show a dose-dependent decrease in cell viability after palbociclib treatment. This is in line with other in vitro studies showing that pan-CDK or specific CDK4/CDK6 inhibition in osteosarcoma resulted in growth inhibition of cells and increased senescence and/or apoptosis^39,40^.

In the current study, 2D cultures were more sensitive compared to 3D cultures. The difference in sensitivity is not surprising and could be explained by the formation of tight intercellular contacts within MCTS which may hamper drug penetration^41^. In general, the concentrations of palbociclib used in the current study, especially in 3D, are higher compared to concentrations used in the clinic for treatment of HER-2 negative breast cancer and in breast cancer cell lines in vitro^42^. However, we have treated the cells with a single dose, whereas in the clinic multiple doses are administered. Moreover, the IC_50_ values determined in the current study are in a comparable concentration range when compared to other in vitro studies in osteosarcoma cells using palbociclib^39,40^. Thus, our data support previous findings that CDK4/CDK6 inhibition might be a new targeted treatment strategy for osteosarcoma patients^40,43^. However, more research is needed to investigate the efficacy of palbociclib for osteosarcoma patients, and whether there are any adverse effects. For the treatment of breast cancer, it was reported that the adverse effects of palbociclib include neutropenia, leukopenia or anaemia^42,44^.

Currently, two phase 2 clinical trials are ongoing which include advanced cases of sarcoma that overexpress CDK4, including osteosarcoma, for palbociclib treatment or similar CDK4/CDK6 inhibitors^45,46^. One of these studies was also designed to test the utility of CDK4 expression in predicting tumour response to CDK inhibitors, but this study did not evaluate whether patients show loss of p16^INK4A^ or intact Rb. In the current study sensitivity to palbociclib did not correlate with CDK4 expression in osteosarcoma cells.

Not only CDK4, but also CDK6 was evaluated as a prognostic and predictive biomarker in our study. Interestingly, about half of the osteosarcoma patients (44.8%) showed overexpression of CDK6 which was associated with a worse overall survival. In this group of patients palbociclib could be beneficial to improve outcome. Palbociclib is most effective when Rb is intact, which was the case in approximately half (52.9%) of the patients with CDK6 overexpression. The expression status of CDK4 and CDK6 is important to consider, since it was recently published that palbociclib selectively dissociates p21 from cyclin D1-CDK4-p21 complexes and not of cyclin D1-CDK6-p21 complexes, which could affect drug sensitivity and resistance^47^.

Another subgroup of osteosarcoma patients that might benefit from CDK4/CDK6 inhibition, include patients with intact Rb, but loss of p16 expression. The in vitro study confirms that osteosarcoma cell lines with intact Rb and/or loss of p16 are more sensitive to palbociclib compared to osteosarcoma cell lines with loss of Rb and/or intact p16. Furthermore, osteosarcoma cell lines with loss of p16 staining by immunohistochemistry showed a trend towards higher sensitivity to palbociclib compared to cells without loss of p16. The immunohistochemical study on the tissue microarray indicates that 13.3% of the patients falls within this category of intact Rb and loss of p16 that may benefit from palbociclib treatment. Taken together, our results illustrate that there is a clear subset of osteosarcoma patients (20–23%) for which CDK4/CDK6 inhibition might be promising and that loss of p16 protein expression or overexpression of CDK6, combined with intact Rb, may serve as a biomarker to select eligible patients.

In conclusion, this study demonstrated that our model of transformed murine MSCs provide a valuable tool to identify targets for therapy and the identification of biomarkers for osteosarcoma patients. Our results illustrate that loss of *CDKN2A* and/or *CDKN2B* are early events in the development of osteosarcoma, and that these events can be targeted by CDK4/CDK6 inhibition, which might be used as a novel therapeutic option in approximately 20–23% of the patients.

## Supplementary information

All supplemental figures

## Data Availability

All data generated or analyzed during this study are included in this published article [and its supplementary information files].
