# Supplementary material for: A murine mesenchymal stem cell model for initiating events in osteosarcomagenesis points to CDK4/CDK6 inhibition as a therapeutic target
Source: Lab Invest. 2021 Dec 17;102(4):391–400. doi: 10.1038/s41374-021-00709-z (PMC8964417; doi:10.1038/s41374-021-00709-z)
Supplement: Supplementary file 1 — All supplemental figures [file 41374_2021_709_MOESM1_ESM.pdf]

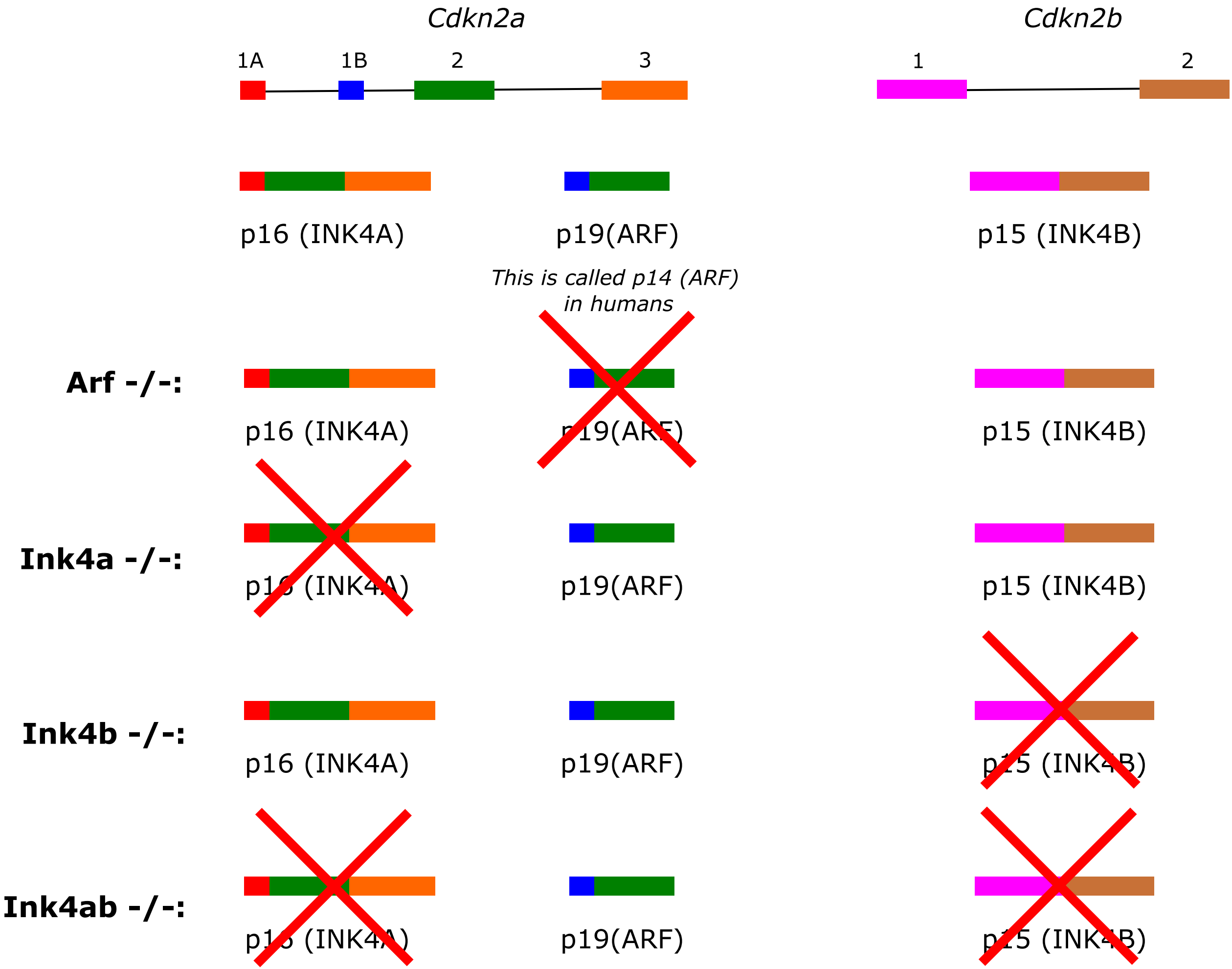

Supplementary Figure S1. Schematic overview of the *Cdkn2a* and *Cdkn2b* locus, and the different KO mice used in this study

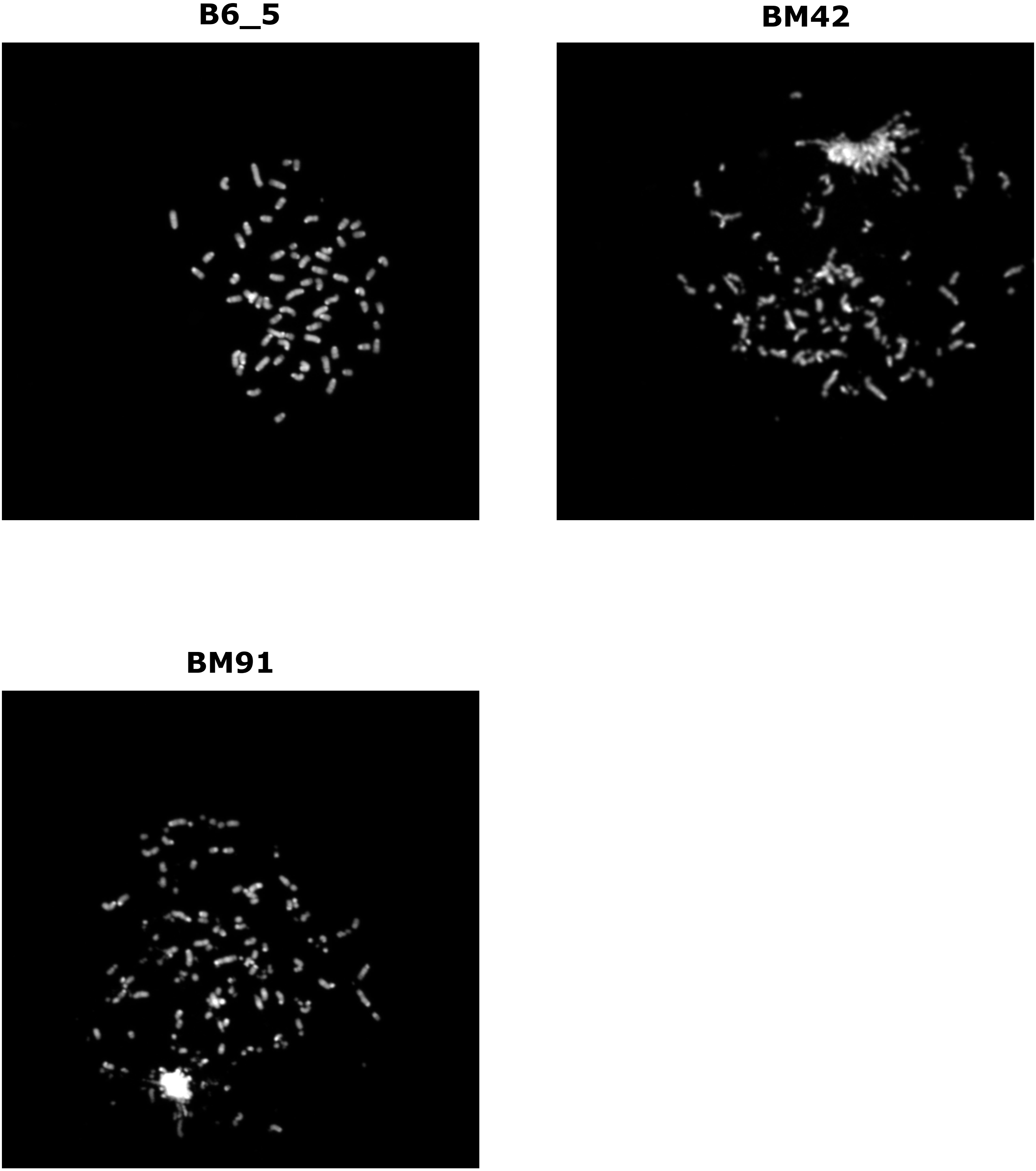

Supplementary Figure S2. Metaphase karyotyping of murine MSCs from B6_5, BM42 and BM91 mice showed abnormal chromosome numbers, higher than the normal modal number of 40.

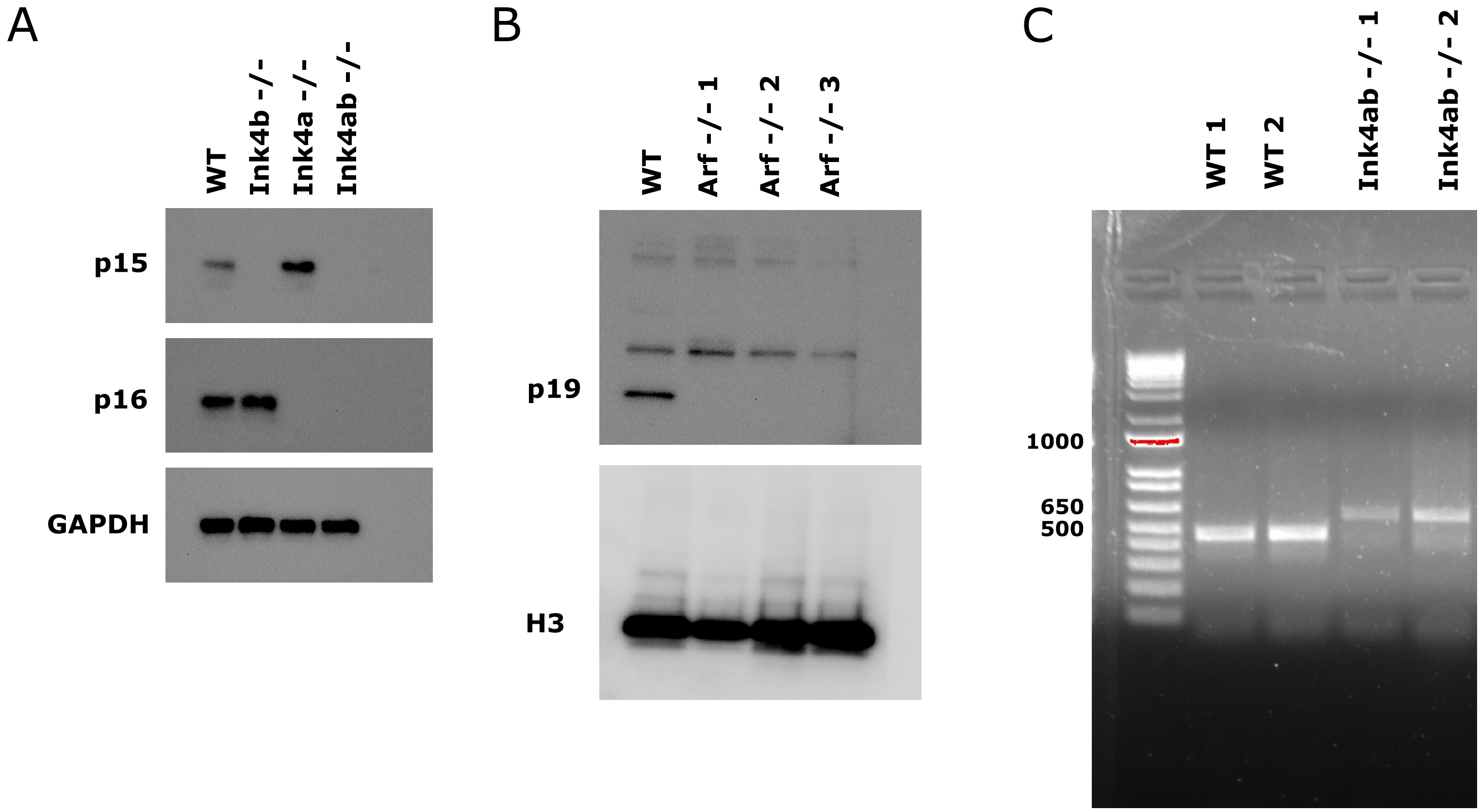

Supplementary Figure S3. Confirmation of knock-out of murine MSCs. Western blots showing (**A**) p15, p16 and (**B**) p19 protein expression of KO MSCs. GAPDH or Histon H3 were used as a loading control. (**C**) DNA electrophoresis gel confirmed KO in MSCs from Ink4ab$_{-/-}$ mice. PCR with primers flanking the lox sites results in a product of 464 bp for the wildtype allele, and 574 bp for the Ink4ab -/- allele

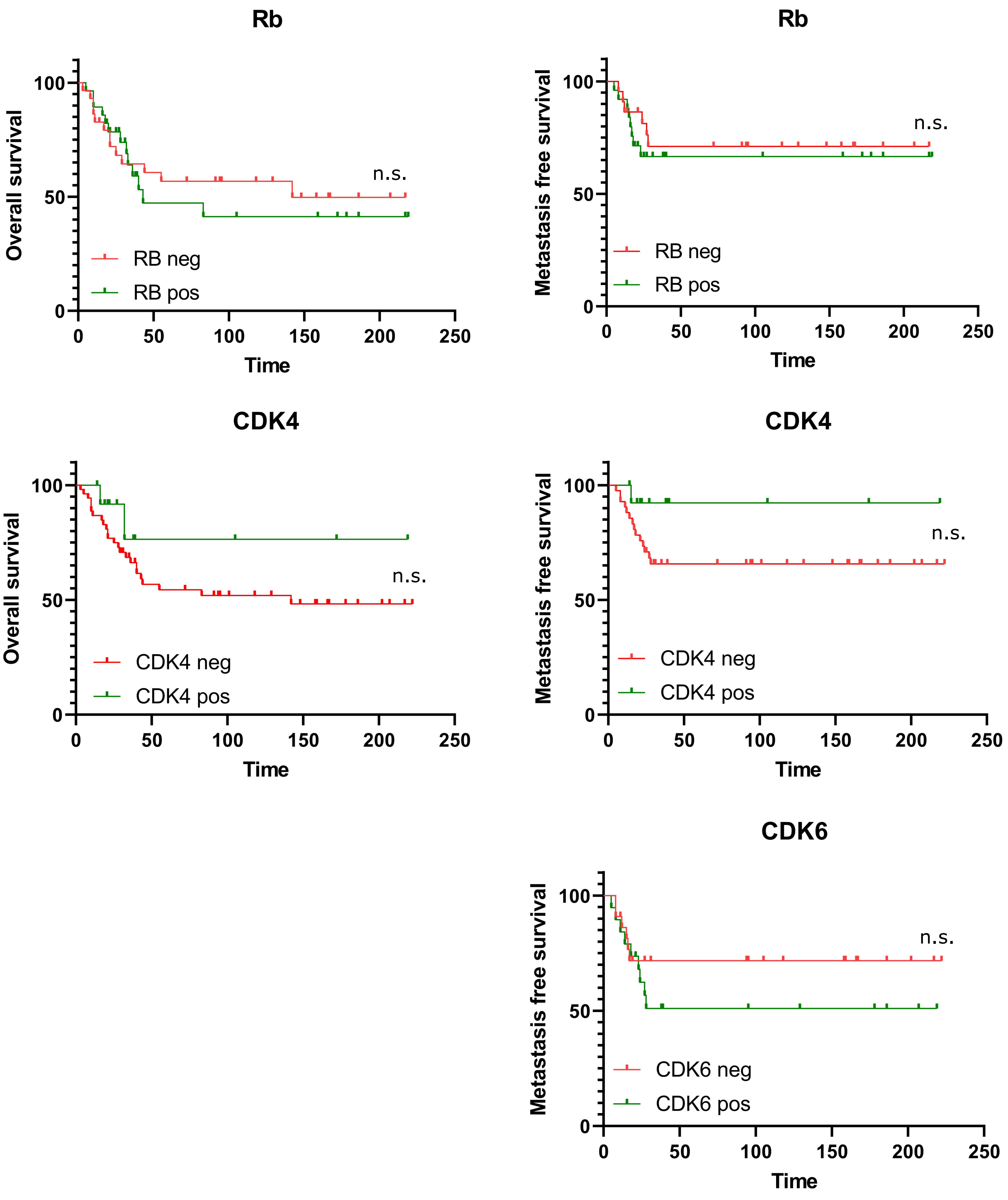

Supplementary Figure S4. Kaplan-Meier curves of overall survival and metastasis-free survival, based on Rb, CDK4, or CDK6 scores.

	Cohort 1	Cohort 2	Cohort 3	Cohort 4	Total
**Total patients**	**14**	**88**	**39**	**17**	**158**
**Total samples**	23	131	69	33	**257**
Biopsies of primary tumour	4	63	27	15	**109**
Resections of primary tumour	8	44	23	17	**93**
Local recurrence	6	0	3	1	**10**
Metastatic lesions	5	24	16	0	**45**
**Year of diagnosis**	1998-2011	1984-2003	2003-2009	2016-2019	**1984-2011**
**Median follow up (months)**	54	98	32	28	**71**
**Number of patients with follow-up data**	13	87	38	17	**155**
**Location of primary tumour**					
Femur	9	45	22	12	**88**
Tibia/fibula		31	9	3	**43**
Humerus	3	10	4	2	**19**
Costa		1			**1**
Hand		1			**1**
Distal radius	1		2		**3**
Os ilium			1		**1**
Os temporale			1		**1**
Other	1				**1**
**Histological response to pre-operative chemotherapy in primary tumour**					
Unknown	4	12	4		**20**
Poor response (<90% necrosis)	8	48	21	9	**86**
Good response (≥90% necrosis)	2	28	14	8	**52**
**Sex**					
Male	11	47	23	13	**94**
Female	3	41	16	4	**64**
**Age at diagnosis**					
Age range	(10-70)	(5-45)	(6-38)	(10-34)	**(5-70)**
Average	34	15	17	16	**17**
Median	32	16	15	15	**15**
**Histotype**					
Osteoblastic conventional OS	4	59	10	11	**84**
Chondroblastic conventional OS	2	9	4	2	**17**
Fibroblastic conventional OS		3		1	**4**
Juxtacortical OS	1				**1**
Small cell OS			1		**1**
Unknown	7	8	26	3	**44**

Supplementary Table S1. Clinicopathological data of tissue micro arrays.
